# Ferroptosis in microglial activation: a systematic review and multidata comparison

**DOI:** 10.1093/braincomms/fcag109

**Published:** 2026-03-30

**Authors:** Ida Pesämaa, Srinivas Koutarapu, Henrik Zetterberg, Stefanie Fruhwürth

**Affiliations:** Department of Psychiatry and Neurochemistry, Institute of Neuroscience and Physiology, the Sahlgrenska Academy at the University of Gothenburg, Gothenberg 413 45, Sweden; Department of Pathology and Immunology, Washington University School of Medicine, St Louis, MO 63110, USA; Department of Psychiatry and Neurochemistry, Institute of Neuroscience and Physiology, the Sahlgrenska Academy at the University of Gothenburg, Gothenberg 413 45, Sweden; Clinical Neurochemistry Laboratory, Institute of Neuroscience and Physiology, Sahlgrenska University Hospital, Mölndal 431 80, Sweden; Department of Neurodegenerative Disease, University College London (UCL) Institute of Neurology, London WC1N 3BG, UK; UK Dementia Research Institute at University College London, London NW1 3BT, UK; Department of Pathology and Laboratory Medicine, University of Wisconsin School of Medicine and Public Health, Madison, WI 53705, USA; Wisconsin Alzheimer’s Disease Research Center, School of Medicine and Public Health, University of Wisconsin, University of Wisconsin-Madison, Madison, WI 53792, USA; Department of Psychiatry and Neurochemistry, Institute of Neuroscience and Physiology, the Sahlgrenska Academy at the University of Gothenburg, Gothenberg 413 45, Sweden

**Keywords:** microglia, ferroptosis, inflammation, neuroinflammation

## Abstract

Ferroptosis is a redox-driven and iron-dependent type of programmed cell death, with lipid peroxidation as a central and required feature of the process. During ferroptosis, cells exert strong proinflammatory effects, suggesting that ferroptosis may play a role in the regulation of inflammation and immune response. However, very few studies have investigated the process of ferroptosis and lipid peroxidation in microglia, the innate immune cells of the brain. In this review, we summarize the concept of ferroptosis and present a list of 120 ferroptosis-relevant proteins, which includes over twice as many entries as the current Kyoto Encyclopaedia of Genes and Genomes (KEGG) pathway for ferroptosis. We compare our manually compiled list with microglial activation signatures reported by us and others, revealing ferroptosis-relevant changes in models for microglial activation. Finally, we highlight a selection of ferroptosis-relevant proteins as potential biomarker candidates for ferroptosis.

## Teaser

In this review, Pesämaa *et al*. collect and present evidence of ferroptosis-related pathways in activated microglia. They compile an expanded list of ferroptosis-relevant proteins and compare it with reported microglial activation signatures, highlighting extracellular detectability and proposing that ferroptosis warrants further investigation as a potential contributor to microglial biology and biomarker development.

## Graphical Abstract

**Graphical Abstract:**
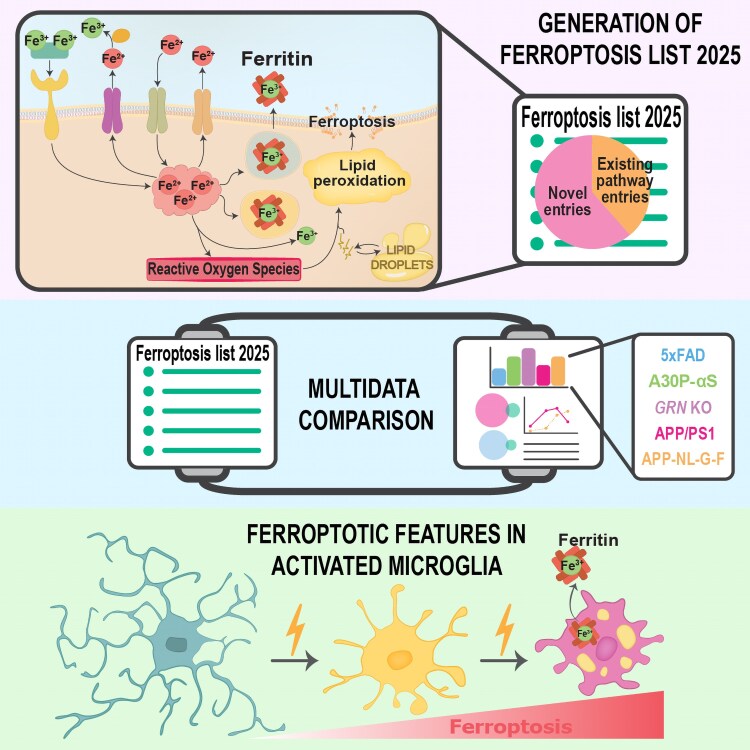

## Introduction

Cell death is an irreversible physiological process occurring when cells are incapable of maintaining their most fundamental functions. Cell death modalities are traditionally classified as an uncontrolled process (non-programmed cell death) or as an orchestrated event involving signalling cascades (programmed cell death). Programmed cell death may be further categorized as lytic (e.g. necroptosis and pyroptosis) or non-lytic (e.g. apoptosis). While apoptosis is generally regarded as immunologically silent, lytic forms of cell death provoke inflammatory responses through the release of proinflammatory signals.^1-3^

The term ferroptosis was first coined in 2012 to describe a redox-driven and iron-dependent type of programmed cell death with proinflammatory properties.^4^ The proinflammatory features of ferroptosis include the release of cytokines (e.g. TNFα, IL-1β, IL-6, CXCL1, CXCL8 and GM-CSF) and profound alterations of lipid metabolism culminating in extensive lipid peroxidation.^5,6^ Products of lipid peroxidation may act as proinflammatory mediators and, in parallel, compromise the integrity and function of the plasma membrane.^5^ Morphologically, ferroptosis is largely associated with mitochondrial abnormalities, including mitochondrial shrinkage and increased mitochondrial membrane density. In contrast to apoptosis, ferroptosis predominantly involves plasma membrane rupture, consistent with its classification as a lytic process.^7^ Nevertheless, ferroptotic features have also been observed in the absence of plasma membrane rupture, with the potential for intercellular propagation through direct membrane contacts.^8^ Together, these features highlight ferroptosis as a uniquely multifaceted cell death programme with mechanistic diversity and biological complexity.

In this review, we summarize the main features and pathways of ferroptosis with a particular focus on neurodegenerative diseases and microglial biology. We also present our manually curated list of 120 ferroptosis-relevant proteins, which we compare with microglial activation signatures reported by us and others across multiple models where microglial activation has been confirmed: (i) 5xFAD mouse microglia,^9^ (ii) *Grn* knockout (ko) mouse microglia and CSF,^10^ (iii) *GRN* ko human induced pluripotent stem cell-derived microglia (hiMG) lysate and media,^10^ (iv) APP/PS1 mouse microglia,^11^ (v) APP-NL-G-F (APP-KI) mouse microglia^11^ and (vi) A30P-αS mouse CSF.^12^ These comparisons demonstrate both the presence of ferroptotic features in activated microglia and the extracellular detectability of ferroptosis-related proteins. Together, these observations indicate that ferroptotic processes are active within microglia and, despite the complexity and need for further mechanistic investigation, can be measured extracellularly. This highlights a key opportunity for biomarker research to define and evaluate the ferroptotic profile of microglia.

## Iron homeostasis and the brain

**Figure 1:**
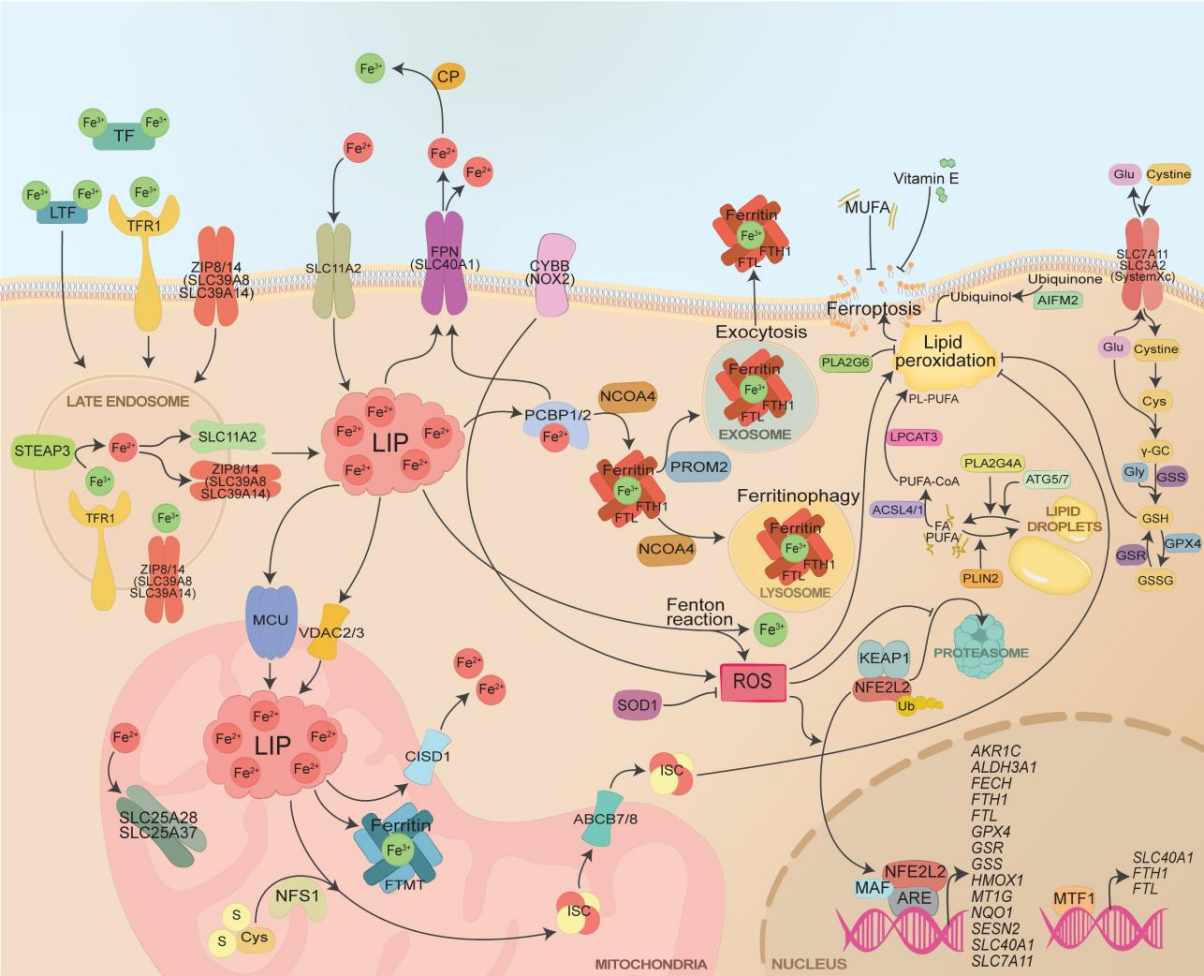
**An overview of ferroptosis.** Graphical illustration summarizing ferroptosis-related pathways with the associated proteins (denoted by their gene name) and genes. This diagram integrates data from various sources and methodologies, providing a generalized representation of ferroptosis-relevant mechanisms that are not specific to any cell type or species. ROS, reactive oxygen species; LIP, labile iron pool; FA, fatty acid; PUFA, polyunsaturated fatty acid; MUFA, monounsaturated fatty acid; ISC, iron-sulphur cluster; ARE, antioxidant response elements.

In the brain, both ferrous (Fe^2+^) and ferric (Fe^3+^) iron, is involved in many different processes such as myelination, neurotransmission, oxygen transport, cellular division and mitochondrial energy production.^13-16^ Iron homeostasis is regulated by several factors involved in the import, export and storage of iron. Moreover, the maintenance of iron equilibrium also depends on the dynamic conversion of Fe^2+^ and Fe^3+^, a process which is regulated by ferrireductases and ferroxidases. Increased intracellular iron accumulation and expansion of the labile iron pool (LIP) promote excessive formation of reactive oxygen species (ROS), leading to toxic downstream effects such as oxidative stress and lipid peroxidation (Fig. 1).^17,18^ Lipid peroxidation is a distinct feature and promoter of ferroptosis.^19,20^ In addition to intracellular iron accumulation and lipid peroxidation, typical hallmarks of ferroptosis include increased production of autophagosomes, cytoplasmic swelling, impaired membrane integrity and mitochondrial abnormalities due to ROS.^21^

**Figure 2:**
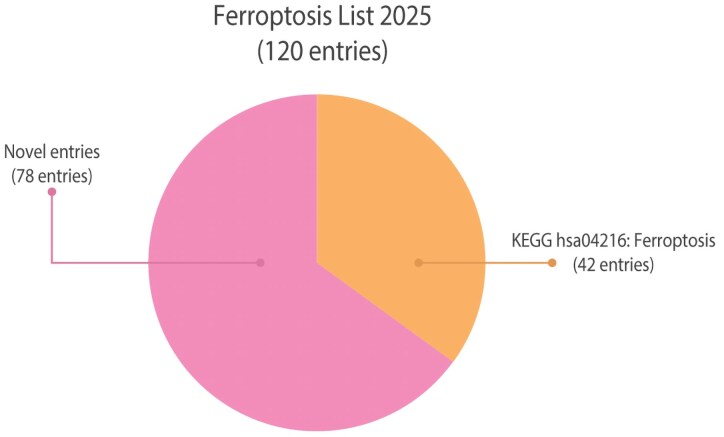
**Ferroptosis list 2025: a manually generated list of ferroptosis-associated proteins.** Our manually curated list of 120 ferroptosis-relevant proteins, comprising 42 proteins annotated in the KEGG ferroptosis pathway and 78 additional unique entries that are absent in the KEGG ferroptosis pathway. Source: KEGG, https://www.genome.jp/entry/pathway+hsa04216; November 28 2025.

Notably, microglia, the innate immune cells of the central nervous system (CNS), have been suggested to encompass the highest iron storage capacity of all the cell types within the brain.^22,23^ However, until recently, the role of ferroptosis in the context of microglial activation had not been studied. Now, accumulating evidence propose that iron and ferroptosis are indeed associated with microglial activation and oxidative stress.^17,24-28^ In this review, we highlight the significance of ferroptosis and lipid peroxidation, emphasizing their association with neurodegenerative diseases and their potential involvement in microglial activation. Furthermore, our curated list of 120 proteins (referred to by gene name), ‘Ferroptosis List 2025’, offers a valuable resource for pathway analysis, particularly given the limited representation of ‘ferroptosis’ in current data bases (with only 42 entries in KEGG pathway hsa04216) (Fig. 2). Lastly, we highlight extracellularly detectable proteins as potential biomarker candidates for assessing ferroptotic signatures.

## The cellular uptake of iron

There are different mechanisms as to how iron is imported into the cell, with the most common being endocytosis-mediated uptake via the transferrin receptor 1 (TFR1, encoded by the *TFRC* gene). TFR1 is a cell surface receptor and an acknowledged marker for ferroptosis.^29^ The iron uptake by TFR1 is enabled by transferrin (TF), a secreted glycoprotein that binds two Fe^3+^ ions before binding to TFR1 and being transferred across the cell membrane via clathrin-mediated endocytosis (Fig. 1).^29-32^ TFR1 also enables the transfer of TF-bound iron across the blood brain barrier (BBB) and into the CNS.^33,34^ However, under conditions such as injury, ischaemia, inflammation or ageing, non-TF-bound iron (NTBI) and ferritin may also cross the BBB via lipid raft domains enriched in the scaffolding protein caveolin-1 (CAV1), which plays an important role in transcellular transport as well as in lipid homeostasis.^32,35,36^ While TFR1 and CAV1 mediate the transport of iron from the blood into the CNS, hepcidin (encoded by the *HAMP* gene) counteracts this event and reduces brain iron levels. The astrocyte-derived hepcidin directly affects the microvascular endothelial cells via the iron exporter ferroportin (FPN, also known as FPN1, encoded by the gene *SLC40A1*).^37,38^

Within the endosome, the acidic environment promotes the release of Fe^3+^, whereupon the non-bound Fe^3+^ is converted to Fe^2+^ by reductases such as six-transmembrane epithelial antigen prostate 3 (STEAP3, also known as TSAP6). Fe^2+^ is then translocated from the endosome to the cytosol via transporters such as DMT1 (encoded by the gene *SLC11A2*). DMT1 is a significant contributor to the cytosolic levels of Fe^2+^, as this transporter is also located at the cell surface where it mediates the translocation of extracellular Fe^2+^ across the plasma membrane into the cytosol (Fig. 1).^21,39-41^ Similar to TF, lactotransferrin [LTF, also known as lactoferrin (LF)] binds extracellular Fe^3+^ and promotes ferroptosis by increasing the cellular uptake of iron.^42^

In addition to DMT1, NTBI may be taken up by metal cation symporters Zrt- and Irt-like protein 8 (ZIP8) and ZIP14 (encoded by genes *SLC39A8* and *SLC39A14*, respectively). ZIP8 and ZIP14 mediate the uptake of NTBI across the plasma membrane, as well as the release of Fe^2+^ from the endosome (Fig. 1).^43-46^ Recently, another member of the ZIP family, namely ZIP7 (encoded by the *SLC39A7* gene), has been proposed as a novel genetic factor associated with ferroptosis.^47^

## Lipid peroxidation a key feature of ferroptosis

Within the cell, Fe^2+^ (as present in the LIP) may be processed and encounter different fates. One fate of Fe^2+^ is the Fenton reaction, where Fe^2+^ reacts with H_2_O_2_ to yield Fe^3+^ and highly reactive hydroxyl radicals (HO^•^). While Fe^3+^ is less reactive, the resulting ROS can cause oxidative stress and subsequent damage to DNA, proteins and lipids—including lipid peroxidation (Fig. 1).^48-51^

As a later hallmark of the ferroptotic process, with subsequent membrane rupture as the ultimate endpoint, lipid peroxidation represents a dynamic process that can actively drive ferroptosis through self-amplifying mechanisms. Ferroptotic features can be present without detectable lipid peroxidation (e.g. iron dyshomeostasis and extensive ROS formation), but lipid peroxidation is nonetheless required for ferroptosis to proceed as a canonical cell death modality.^52,53^ Prior to compromising membrane integrity, lipid peroxidation involves the progressive accumulation of lipid peroxides. Thus, lipid peroxidation represents a central, self-reinforcing driver of the ferroptotic cascade.^51,54^ Apart from ROS, fatty acids (FAs), especially polyunsaturated fatty acids (PUFAs), constitute a central component of the process of lipid peroxidation (Fig. 1). A cell’s susceptibility to ferroptosis is largely dependent on the homeostasis of PUFA synthesis and degradation.^30,54^ Accumulating evidence demonstrate the importance of beta-oxidation (a common lipid metabolism pathway), which regulates the availability of FAs in the cytosol. Once beta-oxidation is disrupted there is an increase of cytosolically available FAs, which promotes lipid peroxidation and subsequently ferroptosis.^30,55-57^ PUFAs, especially arachidonic acid, are favourable substrates for lipid peroxidation.^30,56^ The incorporation of PUFAs into membrane phospholipids requires acylation and esterification, reactions that heavily rely on acyl-CoA synthetase long-chain family member 4 (ACSL4) and lysophospholipid acyltransferase 5 (LPCAT3). The final step of lipid peroxidation takes place at the cellular membrane, where the phospholipid-polyunsaturated fatty acids (PL-PUFAs) are attacked by ROS and lipid peroxides are formed (Fig. 1).^30,58^ Interestingly, exogenous monounsaturated fatty acids (MUFAs) may replace the PUFAs within the plasma membrane and prevent lipid peroxidation and thereby also ferroptosis. This protective mechanism is not completely understood, although acyl-CoA synthetase long-chain family member 3 (ACSL3) appear to be involved in the incorporation of MUFAs into the membrane.^59,60^

## Iron homeostasis and anti-ferroptotic components

In addition to the Fenton reaction, other possible fates of intracellular Fe^2+^ include iron storage, the formation of iron-sulphur clusters and non-ROS generating ferroxidation. Intracellularly, Fe^2+^ may interact with ferritin, which is a protein complex consisting of ferritin heavy chain 1 (FTH1) and ferritin light chain (FTL). The main role of ferritin is to store iron and to prevent Fenton reactions, by binding and converting toxic Fe^2+^ to non-toxic Fe^3+^ via ferroxidase activity. A similar iron storage process is present within the mitochondria, with mitochondrial ferritin (FTMT) as the key component.^43,61^ FTMT has been shown to effectively regulate intracellular iron homoeostasis, as ferroptosis models with FTMT overexpression display significant reductions in both LIP and ROS.^62^ By converting redox-active Fe^2+^ to Fe^3+^ and safely storing iron, ferritin and FTMT protect cells from iron-mediated oxidative damage and thereby mitigate their susceptibility to ferroptosis. The ferritin-bound iron is normally either exported via exocytosis or degraded through ferritinophagy, a specific form of autophagy. Autophagic processes, particularly ferritinophagy, play an important role in ferroptosis, as they regulate the cytoplasmic homeostasis of both iron and lipids.^63^ Furthermore, autophagic activity has been reported to promote ferroptosis through the regulation of *SCL7A11* and glutathione peroxidase 4 (GPX4).^63,64^

Intracellular iron homeostasis heavily relies on the cellular export of iron, which is crucial for preventing iron accumulation and mitigating the risk of ferroptosis. Reported as the only known iron exporter, FPN plays a central role in iron homeostasis.^65^ Fe^2+^ is delivered to FPN via poly (RC) binding protein 2 (PCBP2) (Fig. 1).^43,49^ PCBP1 and PCBP2 work as iron chaperons, although the literature mainly covers PCBP2 and its relevance to iron homeostasis by receiving and delivering iron from iron importers (such as DMT1) to exporters (such as FPN).^66^ Prominin-2 (encoded by the *PROM2* gene) is also involved in the export of iron, but in the form of exocytosis (Fig. 1).^21,43^ Following iron export through FPN, iron homeostasis is mainly maintained by ceruloplasmin (CP). CP is an extracellularly located glycoprotein, capable of oxidizing Fe^2+^ to Fe^3+^ without the generation of ROS (Fig. 1).^67^ CP, with its ferroxidase activity, plays a central role in iron homeostasis and is considered protective against ferroptosis.^68^

In addition to increased iron export, ferroptosis may be counteracted by reducing the iron uptake. Heat shock protein beta 1 (also known as HSP27 and HSP28, encoded by the *HSPB1* gene) inhibits iron uptake and prevents ferroptosis as well as lipid peroxidation. As a result, increased expression of *HSPB1* is considered protective, while reduced *HSPB1* expression result in the opposite effect and promotes ferroptosis.^69^

Given the central role of lipid peroxidation in ferroptosis, several anti-ferroptotic mechanisms are involved in regulating the availability of cytosolic lipids and their propensity to react with ROS. Lipid storage, including the formation of lipid droplets, limits the availability of FAs and PUFAs within the cytosol and thereby reduces their interaction with phospholipids at the plasma membrane. As a result, lipid storage acts as a protective measure against lipid peroxidation, while lipid droplet degradation promotes lipid peroxidation and ferroptosis (Fig. 1).^21,42,43,70^

## System x_c_^−^ and other antioxidative pathways counteracting ferroptosis

System x_c_^−^ is a cystine/glutamate antiporter, consisting of XCT (encoded by the gene solute carrier family 7 member 11, *SLC7A11*) and MDU1 (encoded by *SLC3A2*). This antiporter mediates the specific exchange of extracellular cystine and intracellular glutamate, and its downstream effectors play a crucial role in inhibiting lipid peroxidation. As a result, system x_c_^−^ is recognized as one of the most notable anti-ferroptotic pathways.^58,71^ Once imported by system x_c_^−^, cystine is reduced to cysteine, which, in combination with glutamate, form gamma-glutamylcysteine (γ-GC). Glutathione synthetase (GSS) catalyses the final step, where γ-GC reacts with glycine to yield GSH, the reduced form of glutathione. GSH is an important antioxidant, involved in the detoxification process of ROS as well as in the process of prostaglandin synthesis. GSH is also essential for the enzymatic activity of GPX4, a central regulator of lipid peroxidation and ferroptosis. GPX4 prevents ferroptotic cell death by reducing phospholipid hydroperoxides to non-toxic lipid alcohols.^72^ As the precursor to GSH, γ-GC constitutes a critical factor and a rate-limiting substrate in the process of GSH production, thereby modulating cellular susceptibility to ferroptosis.^73^ In addition to GSS and γ-GC, cytosolic levels of GSH are regulated by the enzyme glutathione reductase (GSR). The oxidized form of glutathione can be converted back into its reduced form (GSH) by GSR, using NADPH as an electron donor. This regeneration is crucial for maintaining the cycle of lipid peroxide detoxification (Fig. 1).^72,74^ The above-mentioned pathways involving system x_c_^−^, GSH and GPX4 can be targeted to trigger and model ferroptosis.^75^

In addition to GSH, ubiquinol is another anti-ferroptotic antioxidant that inhibits lipid peroxidation and ferroptosis via its radical-trapping properties. Ubiquinol is the reduced form of coenzyme Q_10_ (CoQ10). The generation of ubiquinol is dependent on ferroptosis suppressor protein 1 (FSP1, encoded by the gene *AIFM2*), which catalyses the reduction of ubiquinone to ubiquinol at the cell membrane. As a result, both ubiquinol and FSP1 act as inhibitors of lipid peroxidation and ferroptosis (Fig. 1).^60,72^

The transcription factor nuclear factor erythroid 2-related factor 2 (NRF2, also known as NPRF2, encoded by the *NFE2L2* gene), alongside Kelch ECH-associated protein 1 (KEAP1), constitutes a stress-responsive signalling pathway that confers cytoprotective and anti-ferroptotic effects.^76,77^ Under normal conditions, NRF2 is bound to KEAP1, which acts as an adaptor that regulates cytosolic NRF2 levels by enabling ubiquitination and subsequent degradation.^76^ Upon oxidative stress, cysteine residues on KEAP1 become oxidized, resulting in a conformational change that prevents its ability to target NRF2 degradation. As a result, NRF2 accumulates and translocates to the nucleus, where it binds to antioxidant response elements (ARE) in the promoters of target genes. These include a range of anti-ferroptotic and antioxidant genes such as *SLC7A11*, *GPX4*, *SLC40A1*, *HMOX1*, *GSR*, *GSS*, *FTH1*, *FTL*, *NQO1*, *ALDH3A1*, *AKR1C*, *MT1G* and *FECH* (Fig. 1).^21,48^

## Ferroptosis in neurological disorders and diseases

Hemochromatosis, also referred to as iron overload, is characterized by excessive iron accumulation in organs, including the liver, pancreas, heart, joints, skin and pituitary gland. This condition represents the most common genetic disease among individuals of Northern European ancestry and is primarily caused by mutations in the *HFE* gene, which impair the HFE protein’s ability to compete with TF for binding to TFR1, thereby enhancing TF-TFR1 interactions and iron uptake.^78,79^ While hemochromatosis is a systemic disease, neurological symptoms are rare. However, some studies suggest a possible association between hemochromatosis and movement disorders.^80,81^ In contrast, aceruloplasminemia, a rare autosomal recessive disorder caused by (mainly loss-of-function) mutations in the *CP* gene, presents predominantly with neurological symptoms alongside systemic manifestations such as diabetes, retinopathy and liver disease.^82,83^ Notably, aceruloplasminemia has been associated with altered iron homeostasis in glial cells, suggesting a central role for these cells under pathological conditions.^83-85^

Independent of the above-mentioned iron-storage conditions, ferroptosis and iron dyshomeostasis have been linked to several neurodegenerative diseases and neuropathological conditions, including Alzheimer’s disease (AD), Parkinson’s disease (PD), Huntington’s disease (HD), amyotrophic lateral sclerosis (ALS), multiple sclerosis (MS), as well as traumatic brain injury (TBI).^21,22,86-89^ While the exact mechanisms remain unclear, the phenotypes related to ferroptosis and lipid peroxidation seem to depend on the neurodegenerative trigger.

AD is a neurodegenerative disease and the most common form of dementia, where both lipid peroxidation and iron accumulation have been reported in post-mortem brain tissue.^90,91^ A recent study by Thorwald *et al*.^92^ identified significant alterations in ferroptosis-associated proteins, including FTL, FPN and the glutamate-cysteine ligase modifier unit, in post-mortem brain tissue of AD patients compared to controls. Moreover, elevated iron levels in the brain, as observed in post-mortem tissue and by magnetic resonance imaging (MRI), correlated with accelerated cognitive decline in AD.^93,94^ In a biomarker study using the Alzheimer’s disease neuroimaging initiative cohort, cerebrospinal fluid (CSF) ferritin levels were found to correlate with cognitive performance and predict the progression from mild cognitive impairment to AD, further supporting the role of iron in disease pathogenesis.^95^ Notably, treatments with iron chelators (deferoxamine and deferiprone) and antioxidants (ferrostatin-1) have been suggested as potential therapeutic strategies in AD.^16,96^ However, results from a recent phase 2 clinical trial demonstrated that deferiprone treatment was associated with accelerated cognitive decline and an increased risk of neutropenia in patients with early-stage AD.^97^ In addition to these ferroptosis-inhibiting agents, vitamin E is a potent regulator of ferroptosis, which it inhibits through mitigation of lipid peroxidation.^98^ Since 1997, research efforts have explored vitamin E, owing to its antioxidative and anti-inflammatory properties, as a potential AD therapy.^99,100^ Several studies have reported reduced circulating vitamin E levels in AD patients, supporting the hypothesis that vitamin E supplementation may confer therapeutic benefit. However, these findings remain inconsistent, as no clear correlation between AD and vitamin E levels has been observed across studies, and the clinical benefits of vitamin E supplementation in AD remain unreliable.^100^ Nevertheless, attempts to treat AD with vitamin E, especially in combination with other antioxidants, nutrients, or pharmaceuticals, are ongoing and may benefit from a deeper understanding of microglial ferroptosis.

The second most common form of dementia is vascular dementia, which occurs when the blood supply to the brain is obstructed. Vascular dementia may develop after a stroke, which is a common cerebrovascular disease with high mortality and disability rate. In the serum of patients with acute ischaemic stroke high ferritin levels correlate with worse clinical outcomes and increased severity scores.^101,102^ Similar to AD, levels of iron, lipid peroxidation and ferritin are increased in rodents modelling stroke-related brain injuries.^103^ Moreover, post-stroke treatment with a ferroptosis inhibitor (pharmacological selenium) appears to be neuroprotective and rescue brain function in mice.^104^ In addition, complications following intracerebral haemorrhage, which is a severe type of stroke, have been linked to iron-toxicity as resulting from the haemorrhagic event.^105^

In PD, both lipid peroxidation and elevated iron accumulation has been reported.^30,106^ Interestingly, region-specific differences regarding iron accumulation have been reported in early-stage PD (affecting substantia nigra and red nucleus) compared to middle-late-onset PD (affecting substantia nigra and the putamen).^107-109^ A study involving 240 patients with PD, stratified into four groups based on disease severity, identified oxidative stress-induced lipid peroxidation as a key factor in PD progression. Levels of lipid peroxidation products, including lipid hydroperoxides and malondialdehyde, were significantly elevated in plasma at more advanced stages of the disease.^110^ Following a similar rationale to that applied in AD, the antioxidant and anti-inflammatory properties of vitamin E have, since 1999, been considered potentially protective in PD as well. As observed in AD, the therapeutic effects of vitamin E appear variable in PD, with data suggesting that treatment initiation during presymptomatic disease stages may increase the likelihood of therapeutic benefits.^111-114^ In addition to vitamin E, FTH1, with its protective and anti-ferroptotic properties, has recently emerged as a therapeutic candidate in PD, where it regulates ferroptosis through ferritinophagy-related mechanisms.^115^

On a similar note, oxidative stress is markedly elevated in both symptomatic HD patients and asymptomatic gene carriers, and it is considered an early and prominent feature of HD.^116,117^ However, it remains unclear whether it plays a causal role in the disease or arises as a consequence of early pathological events.^116^ Further supporting the involvement of disrupted iron metabolism, increased ferritin accumulation within dystrophic microglia appears to be an early event in HD pathogenesis.^118^ Moreover, brains of R6/2 mice, modelling HD, exhibit abnormal levels of ferroptosis-related proteins. Notably, treatment with the iron chelator deferoxamine improved the motor phenotype in these mice.^119^

Extensive evidence from both ALS models and human biosamples identifies oxidative stress as a critical biological mechanism contributing to disease pathogenesis of ALS.^120^ Superoxide dismutase-1 (encoded by the *SOD1* gene) is an antioxidant enzyme catalysing the breakdown of superoxide radicals, thereby mitigating oxidative stress. Mutations in the *SOD1* gene are recognized as the second most prevalent genetic cause of ALS worldwide. While the underlying mechanisms remain incompletely understood, it is believed that these mutations lead to the formation of misfolded aggregates that contribute to neuronal toxicity and disease progression.^121^ In support of a role for iron dysregulation in this process, studies have reported significantly elevated serum ferritin levels in patients with ALS compared to healthy controls.^122,123^ In another study, plasma ferritin levels rose more rapidly in fast-progressing cases compared to slow-progressing ones, highlighting the potential of iron dysregulation in disease progression.^124^ Moreover, ferroptosis-related features have been linked to microglial activation in ALS models.^125,126^ In primary microglia, isolated from *SOD1* mutant mice, the expression of system x_c_^−^ increased following microglial activation. Additionally, in post-mortem spinal cord tissues from ALS patients, system x_c_^−^ expression appears to correlate with the expression of *CD68*, a marker of activated microglia—further supporting the association between ferroptosis and microglial activation in ALS pathology.^126^

Growing evidence from MRI studies suggest increased accumulation of iron in individuals with clinically isolated syndrome, a condition that often precedes the onset of MS.^127^ Notably, iron-enriched microglia have been observed at the edges of chronic white matter lesions in post-mortem tissue from MS patients.^128^ Consistent with this finding, a separate study reported the presence of iron-laden reactive microglia, along with macrophages, in post-mortem brains of individuals diagnosed with MS.^129^ Further supporting the broader role of iron dysregulation in MS, CSF ferritin levels also appear to be elevated in MS patients compared to controls.^130^ In experimental autoimmune encephalomyelitis (EAE) mice, a widely used animal model to study MS, increased iron levels and lipid peroxidation have been observed.^131^ Preclinical studies involving treatment with lipophilic radical trap compound UMAC-3203 have shown protective effects against early disease progression and a significant delay in relapse onset in EAE mice,^132^ suggesting it as a promising therapeutic target and further highlights the role of ferroptosis in MS pathogenesis.

Superficial siderosis of the CNS is a rare and often underdiagnosed disorder caused by chronic or recurrent bleeding within the subarachnoid space.^133^ This persistent bleeding leads to the accumulation of hemosiderin, an iron storage complex formed from ferritin degradation.^134^ Hemosiderin deposition is particularly harmful, as it progressively results in neurodegeneration.^133^ Notably, hemosiderin deposition has been linked to AD, with detectable accumulations specifically within glial cells.^135,136^ Microglia respond to haemorrhagic injury by upregulating haem oxygenase-1 (encoded by the *HMOX1* gene) to degrade haem and sequester iron, as demonstrated both in animal models and human tissue.^137,138^

TBI, defined as a brain injury caused by external mechanical forces, is clinically classified based on severity into mild (concussion), moderate, or severe cases.^139^ BBB impairment and intracranial haemorrhage due to TBI may permit iron accumulation within the brain tissue, disrupt the iron homeostasis and ultimately contribute to neuronal cell death.^140^ Research using TBI mouse models has demonstrated elevated levels of iron-regulatory proteins, including TFR1, DMT1, GPX4, FPN, FTH1 and FTL, within brain tissue following injury.^141,142^ In human post-mortem brain tissue from individuals who died following stroke or TBI, iron accumulation and increased ferritin expression have been observed within lesioned areas. In the acute phase, these deposits of iron and ferritin are primarily localized within macrophages/microglia, while in chronic or older lesions, astrocytes exhibit ferritin overexpression.^143^ The apparent iron dyshomeostasis following TBI, together with recent findings linking TBI and neurodegenerative diseases, supports the hypothesis that iron dysregulation may play a critical role in potentiating neurodegeneration in TBI-related cases.^144,145^

Taken together, these findings highlight the complex and context-dependent role of iron in neurodegeneration and underscore the need for further investigation into how iron dysregulation contributes to pathological processes, including oxidative stress, cell death, as well as glia-mediated inflammation.

## Ferroptosis and microglia

Microglia are the innate immune cells of the CNS, where they carry out essential functions related to immune surveillance and the maintenance of CNS homeostasis. In a non-activated state, microglia extend motile processes to continuously monitor the CNS environment.^146,147^ When encountering a disturbance such as apoptotic cell debris or other pathological challenges, microglia undergo morphological changes and adopt an activated phenotype. This activated state is characterized by an enhanced phagocytic and lysosomal activity, increased expression of activation receptors, as well as increased secretion of various molecules involved in immune response.^146,148^ It is critical to recognize that microglia are remarkably plastic cells, highly responsive to even slight changes within their environment. As a consequence, their phenotypic states shift significantly in a context- and time-dependent manner.

For phagocytes to fulfil their function and yet survive, careful regulation of iron metabolism, lipid peroxidation and thiol processes is required.^149^ The cytosolic levels of iron-storage protein ferritin differ between cell types and change according to the demand of iron. In the brain, microglia are the cell type with highest levels of cytosolic ferritin, which is likely to reflect the large iron-storage capacity in microglia.^14,150^ In line with this, microglia retain more iron than neurones in conditions of iron overload.^22^ However, a long-term accumulation of iron is likely to cause cellular stress, which might explain the reported association between ferritin and dystrophic microglia.^24,118^

In post-mortem AD tissue, iron-laden microglia were commonly observed near Aβ plaques.^151,152^ The intracellular iron was primarily localized in rounded microglia with dilated cell-branches, morphologically identified as activated microglia. Less frequently, iron accumulation was also detected in microglia located further from Aβ plaques, while no iron deposition was observed near neurofibrillary tangles.^151^ Similarly, in secondary progressive MS (SPMS) autopsy samples, ferritin-positive microglia, indicative of high intracellular iron content, clustered around SPMS lesions. These ferritin-positive cells co-localized with CD68, demonstrating an activated phenotype of the iron-laden microglia near SPMS lesions.^131^ Moreover, increasing evidence from various studies has linked ferroptosis-related characteristics with microglial activation in both HD and ALS.^118,125,126^ Notably, primary microglia exposed to ferroptosis-conditioned media demonstrated increased messenger RNA (mRNA) expression and protein secretion of proinflammatory cytokines, including TNFα, IL-6 and IL-1β.^153^

Taken together, both ferroptosis and lipid peroxidation are features of specific microglial activation states observed in various neurological disorders. As a result, microglial ferroptosis has emerged as a promising therapeutic target for modulating neuroinflammation.^154^ Here, we introduce the concepts of ferroptosis and lipid peroxidation as distinct features of microglial activation, and we present a manually compiled list of ferroptosis-relevant proteins and genes, along with a multidata comparison that supports this framework and identifies novel biomarker candidates for a microglia-derived ferroptosis signature.

## Ferroptosis list 2025—a complement to traditional pathway analysis

Prior to conducting the multidata comparison, we manually compiled a list of 120 ferroptosis-relevant entries, referred to as *Ferroptosis List 2025* (Supplementary table 1, Fig. 2). This process began with a comprehensive literature review to identify ferroptosis-related pathways, which were subsequently consolidated into a single unified overview (Fig. 1). When comparing our manually curated list with the current KEGG pathway for ferroptosis (KEGG, https://www.genome.jp/entry/pathway+hsa04216; 28 November 2025) we identified 78 entries that are not included the KEGG pathway (Fig. 2). To address microglial relevance, a targeted literature search was conducted for each of the 120 entries in our list (via PubMed, search criteria: [gene name] + ‘microglia’; 17 June 2025) and found supporting references for 91 entries (Supplementary table 1).

## Multidata comparison: ferroptosis-related changes identified in activated microglia

In this multidata study, we utilized data from three distinct studies, covering the microglia transcriptome and proteome across four different mouse models: 5xFAD,^9^  *Grn* ko mice,^10^ APP/PS1^11^ and APP-KI.^11^ All studies were conducted independently, with analytical and technical details provided in the respective original publications. As this review includes multidata comparisons, no new data were generated and no additional statistical analyses were performed. To our knowledge, none of the original studies accounted for potential sex differences; therefore, this factor was not considered in our multidata comparison. In brief, microglia from the 5xFAD model were isolated and analysed using transcriptional single-cell sorting,^9^ whereas microglia from *Grn* ko, APP/PS1 and APP-KI mice were isolated by magnetic-activated cell sorting and subjected to discovery proteomic analysis using mass spectrometry.^10,11^ These data were selected based on their confirmed microglial activation profiles and the availability of the data.

First identified through single-cell RNA sequencing in the 5xFAD mouse model, the so-called disease-associated microglia (DAM) signature^9^ has become a widely recognized framework for describing microglial activation. The 5xFAD, APP/PS1, and APP-KI mice all serve as models for amyloid pathology, which is a well-known trigger for microglial activation. In 5xFAD mice, amyloid aggregations are detectable as early as 1.5 months of age,^155^ which closely parallels observations in the APP/PS1 mice,^156^ while APP-KI mice exhibit amyloid aggregation around 2 months of age.^157^ In these amyloid-burden models, microglial activation is initiated extracellularly by external stimuli (accumulation of toxic amyloid species), whereas in *Grn* ko mice, microglial activation arises internally due to intracellular stress as induced by lysosomal impairment.^158,159^ In addition to the data obtained from mice, this comparison includes data from *GRN* ko human induced pluripotent stem cell-derived microglia (hiMG) (Supplementary Fig. 1).^10^

**Figure 3:**
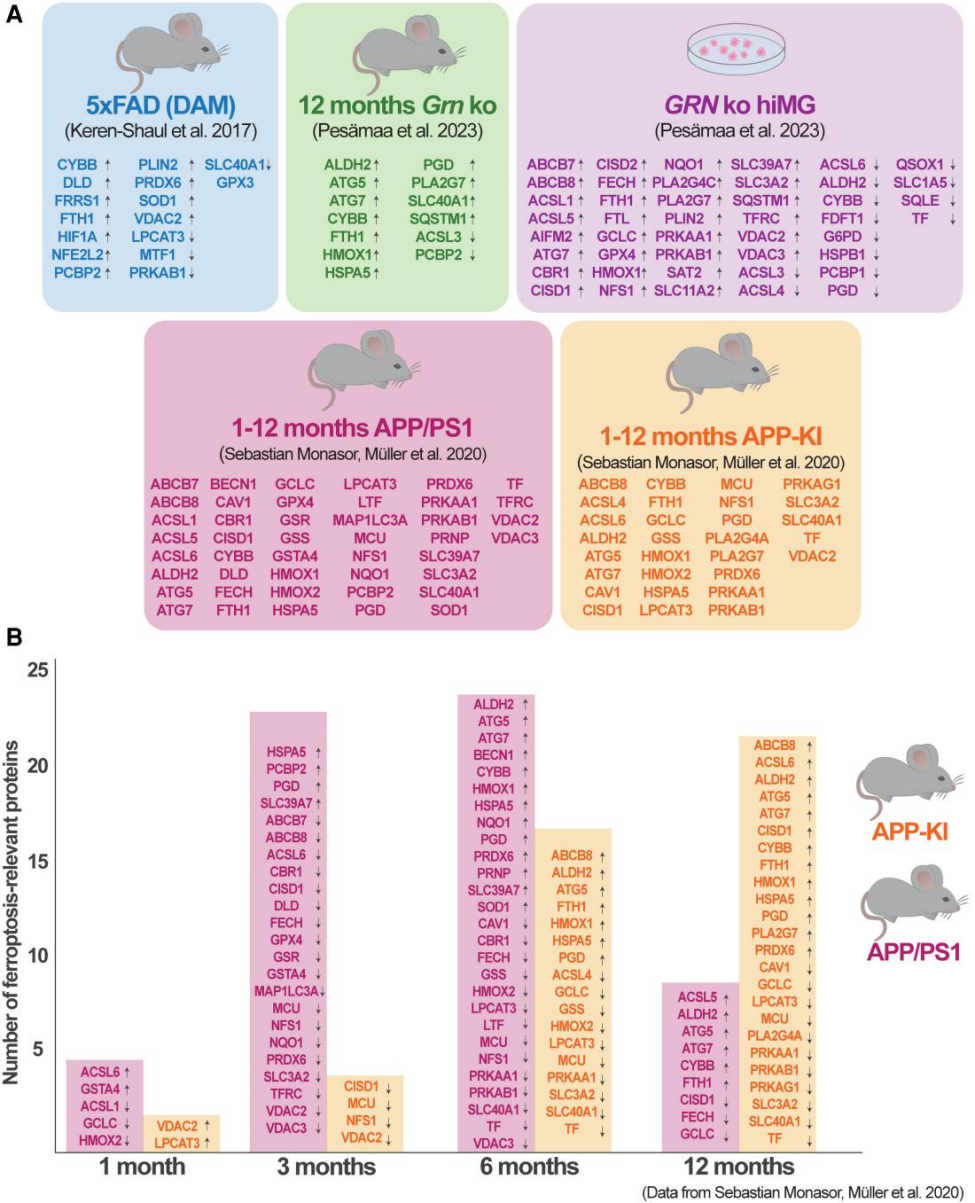
**Ferroptosis-associated biomolecules are significantly altered in activated microglia.** (**A**) Significantly altered levels of ferroptosis-relevant genes and proteins in microglia isolated from models using various means of activation. ‘5xFAD (DAM)’ depicts significant changes of the microglial transcriptome, while ‘12 months *Grn* ko’ and ‘*GRN* ko hiMG’ depict significant changes of the microglial cell proteome. Arrows indicate the direction of change. ‘1–12 months APP/PS1’ and ‘1–12 months APP-KI’ represent summaries of significantly changed proteins, in microglia, at four different ages of the respective mouse model. (**B**) Significantly altered levels of ferroptosis-associated proteins in microglia isolated from APP/PS1 mice and APP-KI mice at ages 1, 3, 6 and 12 months. Arrows indicate the direction of change.

We compared significant alterations (*P*-value < 0.05, excluding values with no assigned gene name) identified in these datasets with our manually compiled list of 120 ferroptosis-relevant proteins (Fig. 2, Supplementary table 1), which revealed several ferroptosis-relevant changes in activated microglia (Fig. 3, Supplementary Fig. 2). Notably, FTH1, a key component of the iron storage complex ferritin, was consistently increased in microglia across all models. Additionally, microglial levels of ferroportin (*SLC40A1*) and PCBP2, both critical for intracellular iron export, exhibited significant alterations in most datasets (Fig. 3)—further highlighting ferroptosis as an important feature of microglial activation.

Other notable changes include autophagy-related gene 5 and 7 (ATG5 and ATG7, respectively), which were significantly increased in all proteomics-based datasets. Specifically, ATG5 and ATG7 were elevated in *Grn* ko mice, APP/PS1 mice and APP-KI mice, whereas only ATG7 was altered in *GRN* ko hiMG (Fig. 3A). Interestingly, in the amyloid-burden mice, these proteins were significantly upregulated following amyloid deposition at 6 and 12 months (Fig. 3B). Peroxiredoxin-6 (encoded by the *PRDX6* gene), which counteracts lipid peroxidation,^160,161^ was significantly changed in microglia isolated from all amyloid models (5xFAD, APP/PS1 and APP-KI) (Fig. 3A).

## Potential biomarker candidates for the evaluation of ferroptosis in the context of neurodegenerative diseases and glial activation

**Figure 4:**
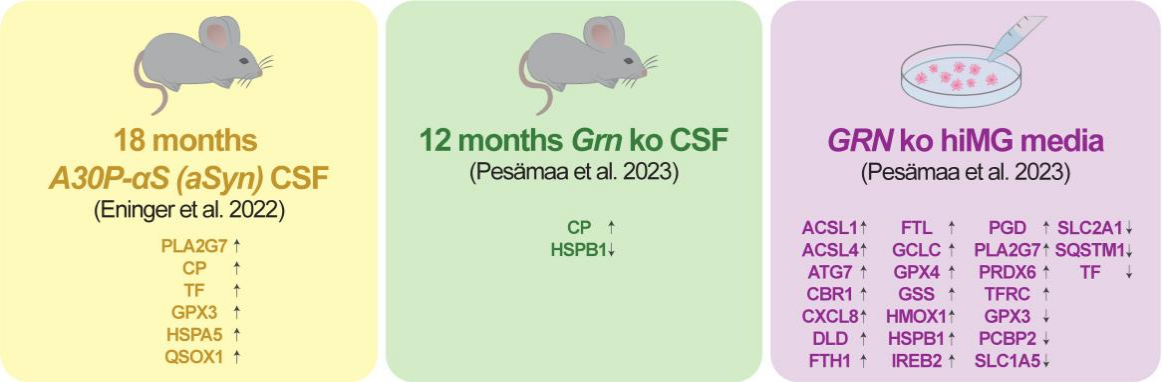
**Ferroptosis-relevant proteins in biofluids of models with confirmed glial activation.** Significantly altered levels of ferroptosis-relevant proteins in biofluids of models with confirmed glial activation: CSF from 18 months old A30P-αS mice, CSF from 12 months old *Grn* ko mice and media from *GRN* ko hiMG. Arrows indicate the direction of change.

The final phase of our multidata comparison incorporated three additional proteomic datasets: CSF from A30P-αS (α-synuclein burden mice with confirmed glial activation), CSF from *Grn* ko mice and conditioned media from *GRN* ko hiMG.^10,12^ To investigate the potential of ferroptosis-associated biomarkers, we compared the proteins with significantly altered levels in these fluids to our manually compiled list of 120 ferroptosis-relevant proteins and genes. In the CSF of A30P-αS mice, six proteins overlapped with our ferroptosis list: PLA2G7, CP, TF, GPX3, HSPA5 and QSOX1. Notably, all six of these proteins showed a significant increase in the CSF of A30P-αS mice compared to control mice (Fig. 4). In the CSF of *Grn* ko mice, only two proteins, CP and HSPB1 aligned with our ferroptosis list, while in the conditioned media of activated hiMG 24 proteins overlapped with our ferroptosis list (Fig. 4). This demonstrates that the detectability of ferroptosis-related proteins in biofluids is possible, and that the levels of these extracellularly detectable ferroptosis proteins are significantly altered following microglial activation (Fig. 4). Among the changes detectable in hiMG media, the levels of ferritin, in the form of both FTH1 and FTL, were significantly increased. Extracellular levels of PLA2G7 were also significantly elevated in the media of activated hiMG, which matches the observed PLA2G7 elevation in CSF of A30P-αS mice (Fig. 4).

## Discussion

Microglial activation is increasingly recognized as a spectrum of states, rather than following a binary classification such as ‘resting’ versus ‘activated’, ‘M1’ versus ‘M2’, or ‘homeostatic’ versus ‘DAM’. This shift towards a more nuanced nomenclature reflects the complexity and dynamics of microglial activation.^162^ As a result, it is improbable that a single marker would suffice for accurately assessing microglial activation, emphasizing the need for a comprehensive panel of biomarkers.

In this review, we introduce ferroptosis and lipid peroxidation as features of microglial activation that should be considered when phenotyping microglia. Given the relatively recent discovery of ferroptosis,^4^ it is often underrepresented in pathway databases, limiting its recognition in comparison to broader and more established pathways. To bridge this gap, we compiled a list of 120 ferroptosis-relevant proteins and genes (Supplementary table 1), which includes over twice as many entries as the current KEGG pathway for ferroptosis (Fig. 2). Our list is available to the scientific community to facilitate deeper investigation into ferroptosis-related findings from transcriptomic and proteomic data. However, it is important to emphasize that this list, like any other pathway resource, should be regarded as a tool rather than a definitive result. Any significant overlaps with our ferroptosis list or widely accepted pathway databases should be interpreted thoughtfully, with careful reasoning to contextualize their biological relevance.

For our multidata comparison, we used our *Ferroptosis List 2025* to investigate the presence of ferroptosis and lipid peroxidation in the context of microglial activation. Comparing our list to datasets of activated microglia, we consistently identified ferritin, detected as either FTH1 or FTL, across all models (Fig. 3). As the primary intracellular storage protein, the increased ferritin levels provide compelling evidence that iron homeostasis is altered in activated microglia, independent of the specific activation trigger, which is likely to affect ferroptotic pathways. Given that microglia are exhibit the highest ferritin expression within the brain,^14^ we propose that CSF ferritin levels, previously associated with neurological disorders such as AD,^163^ may serve as a biomarker for a specific form of microglial activation. The increased levels of FTL and FTH1 likely reflect an adaptive cellular response aimed at mitigating iron-induced oxidative stress rather than serving as a driver of ferroptosis. Notably, the secreted levels of FTH1 or FTL increased with microglial activation *in vitro*, but not in the CSF of *Grn* ko mice (Fig. 4). These findings suggest that the release of microglia-derived ferritin, as represented by either FTL or FTH1, increases with microglial activation, a process that may differ between mouse and human as well as over time, as observed in microglia isolated from APP/PS1 and AAP-KI mice (Fig. 3B). Thus, further studies are required to confirm this hypothesis and clarify its broader implications.

Consistently altered in microglia of all mouse models we found ferroportin (*SLC40A1*), which is a key mediator of iron export. Interestingly, while ferroportin levels were increased in proteomic datasets, transcriptomic data showed a significant reduction in its expression (Fig. 3A). This discrepancy is in line with the complex regulation of ferroportin, which is regulated by multiple mechanisms at the levels of mRNA, post-transcriptional processing and post-translational modification.^164^ The consistent increase of the ferroportin protein, together with the observed increase of ferritin, might suggest that microglia in these models adopt an iron recycling-exporting phenotype, as previously reported in macrophages stimulated with M-CSF, IL-4 or glucocorticoids.^165^ In the amyloid mouse models, the protein levels of ferroportin became significantly elevated only after 6 months. This suggests that its involvement may be part of a more sustained activation profile rather than an immediate response, which aligns with the hypothesis that ferritin and ferroportin function cooperatively in response to iron-induced oxidative stress. Notably, no significant changes in ferroportin were observed in the cell lysate or conditioned media of hiMG (Fig. 3A and Fig. 4, respectively). The divergence between mouse and hiMG data may reflect species-specific differences, *in vivo* versus *in vitro* effects, or the inherently dynamic nature of microglial activation and ferroptosis, both of which are likely to be influenced by microglial age and activation stage. Moreover, given the high plasticity of microglia, the type of stimulus (inflammatory, metabolic or proteotoxic), its origin (i.e. exogenous versus endogenous) and its duration are all known determinants of the microglial activation signature and are therefore very likely to also affect the ferroptosis profile within these cells.

In addition to ferritin and ferroportin, significant increases in ATG5 and ATG7 were observed in activated microglia datasets (Fig. 3), potentially linking microglial activation to lipid peroxidation. Both ATG5 and ATG7 are essential for autophagosome formation, which may promote lipid metabolism and enhance the availability of lipid substrates for peroxidation, which sequentially leads to an increased susceptibility to ferroptosis (Fig. 1).^21^ Although the increased levels of ATG5 and ATG7 might indicate an increased susceptibility of ferroptosis, it cannot be concluded that cells exhibiting elevated ATG5/ATG7 levels actually undergo ferroptosis. In the amyloid mouse models, the levels of ATG5 and ATG7 were significantly increased only after 6 months, suggesting that these proteins, similarly to ferroportin, are associated with a sustained activation profile of microglia. Taken together, these findings highlight the critical need to investigate the role of iron homeostasis and lipid peroxidation, particularly the levels of FTH1, FTL, FPN, ATG5 and ATG7, in the context of sustained microglial activation.

Finally, we applied our ferroptosis list as a tool to explore potential biomarkers indicative of microglial activation features associated with ferroptosis and lipid peroxidation. Although the idea of fluid-based biomarkers for assessing iron homeostasis is not novel, the exploration of biomarkers specifically related to microglial ferroptosis has not, to our knowledge, been previously investigated. We identified ferroptosis-related changes in the fluids of all three models: CSF from 18 months old A30P-αS mice, CSF from 12 months old *Grn* ko mice and media from *GRN* ko hiMG (Fig. 4), with the most pronounced effects observed in the media of activated hiMG, where 24 significantly altered proteins overlapped with our ferroptosis list. Notably, we observed a significant increase in ferritin (both FTL and FTH1) in the conditioned media of activated hiMG (Fig. 4), which aligns with the elevated levels observed in the cell lysate of these cells (Fig. 3A). This highlights ferritin as a promising fluid-based proxy biomarker, suggesting elevated extracellular ferritin levels reflect increased intracellular protein expression. Although microglia are the cell type in the brain with the most abundant ferritin expression,^150^ additional studies are necessary to validate whether CSF ferritin reliably reflects microglial activation, given that this protein is also expressed by other cell types in the brain. Furthermore, FTH1 levels did not reach statistical significance in the CSF of A30P-αS nor in the CSF of *Grn* ko mice, and FTL was not detected in either of these datasets. This underscores the need for further investigation into ferritin biology in terms of (i) mouse-human translatability and (ii) differences in turnover dynamics between media from monocultured microglia versus CSF. In addition to ferritin, levels of PLA2G7 were significantly increased in both A30P-αS CSF and *GRN* ko media. Besides its association to ferroptosis, PLA2G7 has been reported to exert proinflammatory functions, via the NLRP3 inflammasome, in macrophages.^166^ ATG7 was significantly increased in the media of activated hiMG, positioning it as another promising candidate for a fluid-based proxy biomarker. Although not significantly altered in the media of hiMG, CP remains of interest for future investigation, as it is significantly increased in the CSF of both mouse models (Fig. 4). In the absence of a clear association with microglial activation, the elevated CP levels in mouse CSF are likely attributable to activated astrocytes.^167^ Although the cellular origin of CP remains unclear, the observed extracellular increase in CP likely reflects an anti-ferroptotic response to iron dyshomeostasis, ferroptosis, or a combination of both. CP exerts anti-ferroptotic effects through iron binding and by converting Fe^2+^ to Fe^3+^ without generating reactive oxygen species.^67,68^ Interestingly, CP also has the capacity to bind copper, thereby introducing considerations of copper homeostasis and highlighting the importance of future CP-focused studies to investigate ferroptosis and cuproptosis in parallel.^168^

Collectively, FTH1, FTL1 and ATG7 (with ATG5 and CP as additional potential candidates) constitute novel, albeit preliminary, fluid-based biomarkers candidates that may, upon further investigation, prove useful in evaluating specific microglial activation profiles related to ferroptosis.

Species-specific differences and the inherent distinction between *in vitro* monoculture media and *in vivo* CSF should be considered when interpreting these findings. *In vivo*, CSF proteins are subjected to clearance, cellular uptake and receptor-mediated interactions across diverse cell types, whereas *in vitro* protein turnover is limited to processes governed solely by the cultured cells, without the influence of systemic or multicellular factors present in the physiological environment. With notable opposing directionality of change, as observed for some of the proteins, their extracellular functions, with respect to the context and environment in which they are present, remain speculative. Nevertheless, the detection of ferroptosis-relevant proteins in the extracellular environment, with significant changes associated with microglial activation, supports the potential of fluid-based biomarkers for monitoring microglial ferroptosis.

The objectives of this review were to provide a comprehensive overview of ferroptosis mechanisms, present an updated list of ferroptosis-relevant proteins and genes and highlight intra- and extracellular ferroptosis-related changes (both pro- and anti-ferroptotic) associated with microglial activation. Of note, the intended purpose of our multidata comparison was to investigate any potential presence of ferroptosis-related changes, intracellularly and extracellularly, in the context of microglial activation. The datasets used for comparison were selected according to the criteria of (i) confirmed glial activation and (ii) the availability of discovery proteomic or transcriptomic data. Unfortunately, publicly available fluid proteomic data from models with confirmed glial activation are very rare and nearly non-existent for *in vivo* models, due to the technically demanding sampling of CSF from animals. It is important to keep in mind that none of the studies from which the data was obtained were designed to study ferroptosis, which may contribute to the variability observed across models. Additionally, the potential presence of other cell death modalities has not been addressed in these studies. As a result, the potential influence of alternative cell death pathways (e.g. apoptosis, necroptosis, pyroptosis, or cuproptosis) cannot be ruled out by our multidata comparison. Given these limitations, this review aims to encourage renewed attention to ferroptosis within microglia and neuroimmunology research, where further studies are critically needed to delineate the complex and dynamic interplay between ferroptosis and microglial activation.

## Supplementary Material

fcag109_Supplementary_Data

## Data Availability

Data sharing is not applicable to this article as no new data were created or analysed in this study. All data utilized for this review are included in the following published articles and their respective supplementary information files: Pesämaa *et al*. 2023 (doi:10.1186/s13024-023-00657-w), Sebastian Monasor, Müller *et al*. 2020 (doi:10.7554/eLife.54083), Eninger *et al*. 2022 (doi:10.1073/pnas.2119804119). Additionally, our manually curated list of ferroptosis-relevant entries is available as Supplementary Table 1. Graphs and illustrations were designed and generated using Adobe Illustrator 2024, Microsoft Excel (version 16.96.1) and DeepVenn (Tim Hulsen 2020). A list of abbreviations is included in Supplementary Table 2.
