# Supplementary material for: Ferroptosis in microglial activation: a systematic review and multidata comparison
Source: Brain Commun. 2026 Mar 30;8(2):fcag109. doi: 10.1093/braincomms/fcag109 (PMC13056721; doi:10.1093/braincomms/fcag109)
Supplement: fcag109_Supplementary_Data [file fcag109_supplementary_data.zip › Supplementary_Figures_and_Legends.pdf]

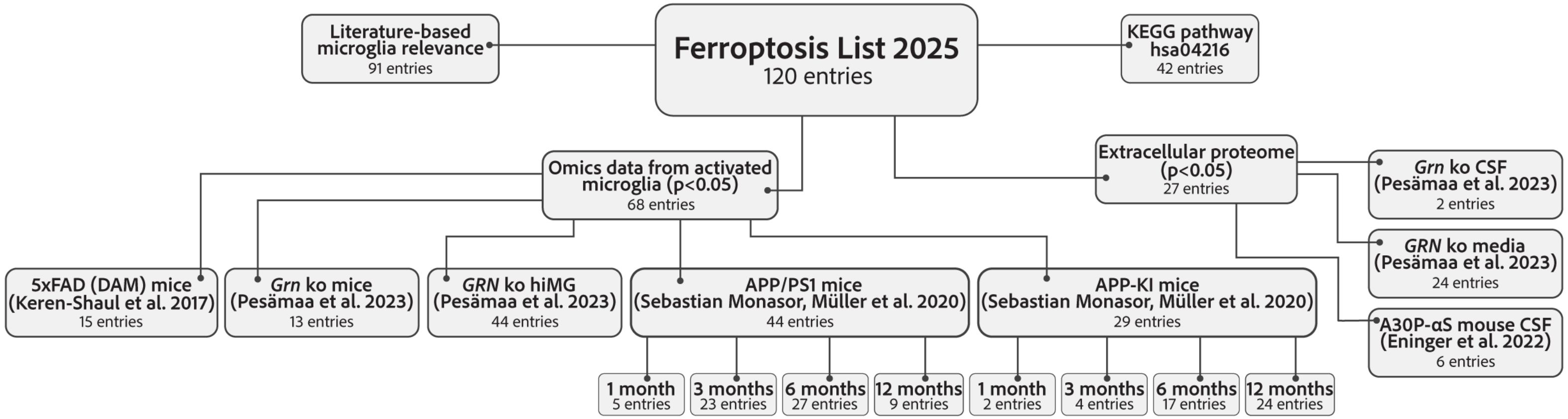

Supplementary figure 1. Overview of multidata comparisons.
Summary of comparisons made against our manually generated list of 120 ferroptosis-relevant proteins, with the respective number of matching entries.

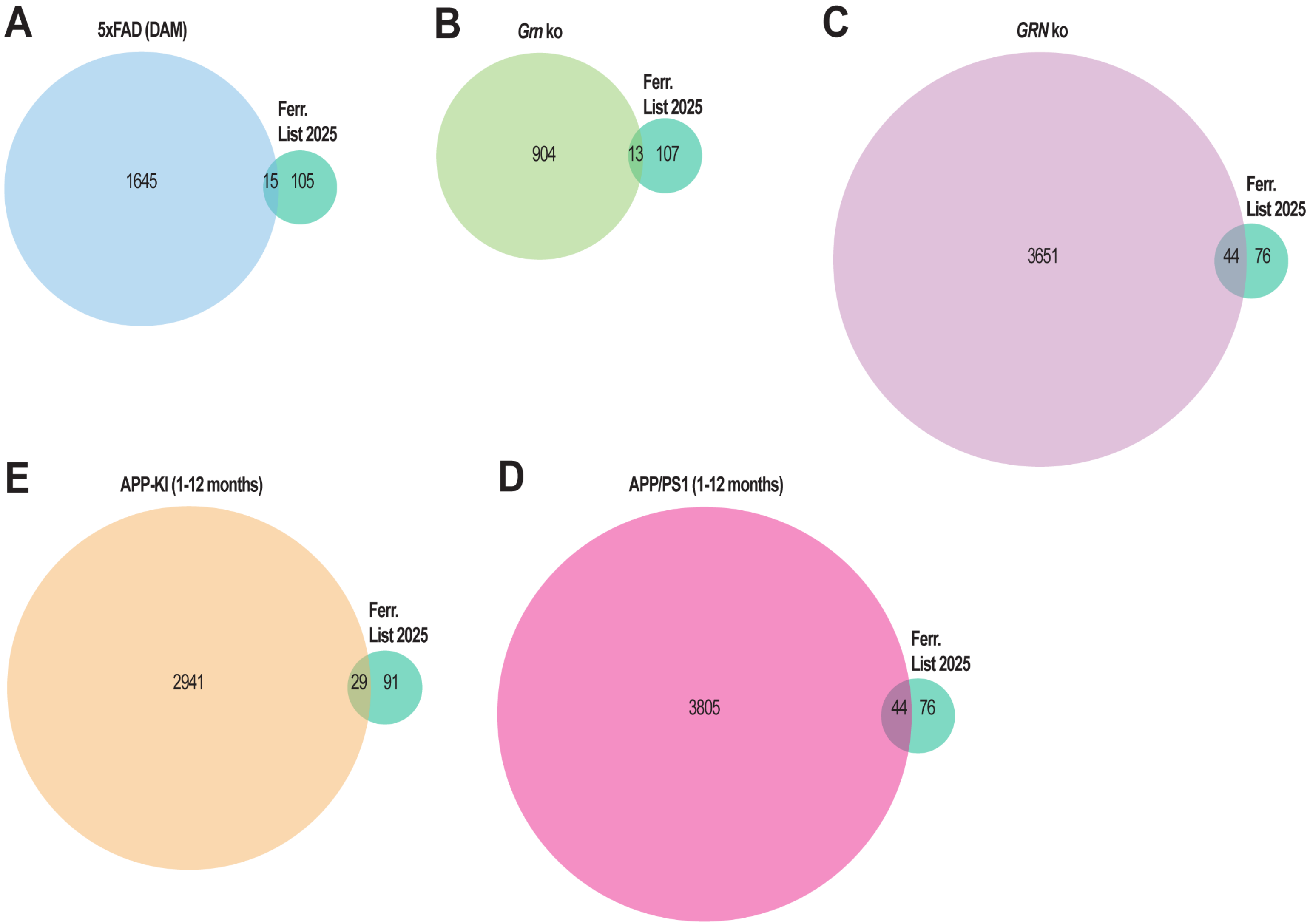

Supplementary figure 2. Ferroptosis-relevant changes in activated microglia.
Venn diagrams representing the overlaps of significantly changed proteins/genes in microglia with confirmed activation, isolated from the following models: (A) 5xFAD mice, (B) Grn ko mice (C) GRN ko hiMG, (D) APP/PS1 mice, and (E) APP-KI mice.
