# Supplementary material for: Ferroptosis in microglial activation: a systematic review and multidata comparison
Source: Brain Commun. 2026 Mar 30;8(2):fcag109. doi: 10.1093/braincomms/fcag109 (PMC13056721; doi:10.1093/braincomms/fcag109)
Supplement: fcag109_Supplementary_Data [file fcag109_supplementary_data.zip › Supplementary_Table_1_References.pdf]

Supplementary table 1 - References

Bartolacci C, Andreani C, Vale G, et al. Targeting de novo lipogenesis and the Lands cycle induces ferroptosis in KRAS-mutant lung cancer [published correction appears in Nat Commun. 2022 Aug 8;13(1):4640. doi: 10.1038/s41467-022-32459-x]. Nat Commun. 2022;13(1):4327. Published 2022 Jul 26. doi:10.1038/s41467-022-31963-4

Battaglia AM, Chirillo R, Aversa I, Sacco A, Costanzo F, Biamonte F. Ferroptosis and Cancer: Mitochondria Meet the "Iron Maiden" Cell Death. Cells. 2020;9(6):1505. Published 2020 Jun 20. doi:10.3390/cells9061505

Beatty A, Singh T, Tyurina YY, et al. Ferroptotic cell death triggered by conjugated linolenic acids is mediated by ACSL1. Nat Commun. 2021;12(1):2244. Published 2021 Apr 14. doi:10.1038/s41467-021-22471-y

Beharier O, Tyurin VA, Goff JP, et al. PLA2G6 guards placental trophoblasts against ferroptotic injury. Proc Natl Acad Sci U S A. 2020;117(44):27319-27328. doi:10.1073/pnas.2009201117

Cao F, Luo A, Yang C. G6PD inhibits ferroptosis in hepatocellular carcinoma by targeting cytochrome P450 oxidoreductase. Cell Signal. 2021;87:110098. doi:10.1016/j.cellsig.2021.110098

Chen P, Jiang Y, Liang J, et al. SLC1A5 is a novel biomarker associated with ferroptosis and the tumor microenvironment: a pancancer analysis. Aging (Albany NY). 2023;15(15):7451-7475. doi:10.18632/aging.204911

Chen PH, Wu J, Xu Y, et al. Zinc transporter ZIP7 is a novel determinant of ferroptosis. Cell Death Dis. 2021;12(2):198. Published 2021 Feb 19. doi:10.1038/s41419-021-03482-5

Chen X, Yu C, Kang R, Tang D. Iron Metabolism in Ferroptosis. Front Cell Dev Biol. 2020;8:590226. Published 2020 Oct 7. doi:10.3389/fcell.2020.590226

Chen Z, Lin H, Wang X, et al. The application of approaches in detecting ferroptosis. Heliyon. 2023;10(1):e23507. Published 2023 Dec 13. doi:10.1016/j.heliyon.2023.e23507

Daimon T, Bhattacharya A, Wang K, et al. MUC1-C is a target of salinomycin in inducing ferroptosis of cancer stem cells. Cell Death Discov. 2024;10(1):9. Published 2024 Jan 5. doi:10.1038/s41420-023-01772-9

Dean M, Allikmets R. Complete characterization of the human ABC gene family. J Bioenerg Biomembr. 2001;33(6):475-479. doi:10.1023/a:1012823120935

Doll S, Freitas FP, Shah R, et al. FSP1 is a glutathione-independent ferroptosis suppressor. Nature. 2019;575(7784):693-698. doi:10.1038/s41586-019-1707-0

Doll S, Proneth B, Tyurina YY, et al. ACSL4 dictates ferroptosis sensitivity by shaping cellular lipid composition. Nat Chem Biol. 2017;13(1):91-98. doi:10.1038/nchembio.2239

Fang X, Wang H, Han D, et al. Ferroptosis as a target for protection against cardiomyopathy. Proc Natl Acad Sci U S A. 2019;116(7):2672-2680. doi:10.1073/pnas.1821022116

Feng F, He S, Li X, He J, Luo L. Mitochondria-mediated Ferroptosis in Diseases Therapy: From Molecular Mechanisms to Implications. Aging Dis. 2024;15(2):714-738. Published 2024 Apr 1. doi:10.14336/AD.2023.0717

Han L, Zhai W. Mechanisms and preventive measures of ALDH2 in ischemia-reperfusion injury: Ferroptosis as a novel target (Review). Mol Med Rep. 2025;31(4):105. doi:10.3892/mmr.2025.13470

Han L, Zhou J, Li L, et al. SLC1A5 enhances malignant phenotypes through modulating ferroptosis status and immune microenvironment in glioma. Cell Death Dis. 2022;13(12):1071. Published 2022 Dec 24. doi:10.1038/s41419-022-05526-w

Hao S et al. (2018). Metabolic networks in ferroptosis (Review). Oncology Letters, 15, 5405-5411. https://doi.org/10.3892/ol.2018.8066

Huang F, Zheng Y, Li X, Luo H, Luo L. Ferroptosis-related gene AKR1C1 predicts the prognosis of non-small cell lung cancer. Cancer Cell Int. 2021;21(1):567. Published 2021 Oct 26. doi:10.1186/s12935-021-02267-2

Ichikawa Y, Bayeva M, Ghanefar M, et al. Disruption of ATP-binding cassette B8 in mice leads to cardiomyopathy through a decrease in mitochondrial iron export. Proc Natl Acad Sci U S A. 2012;109(11):4152-4157. doi:10.1073/pnas.1119338109

Ji HZ, Chen L, Ren M, et al. CXCL8 Promotes Endothelial-to-Mesenchymal Transition of Endothelial Cells and Protects Cells from Erastin-Induced Ferroptosis via CXCR2-Mediated Activation of the NF-κB Signaling Pathway. Pharmaceuticals (Basel). 2023;16(9):1210. Published 2023 Aug 25. doi:10.3390/ph16091210

Kanehisa M, Furumichi M, Sato Y, Kawashima M, Ishiguro-Watanabe M. KEGG for taxonomy-based analysis of pathways and genomes. Nucleic Acids Res. 2023;51(D1):D587-D592. doi:10.1093/nar/gkac963

Kanehisa M, Goto S. KEGG: kyoto encyclopedia of genes and genomes. Nucleic Acids Res. 2000;28(1):27-30. doi:10.1093/nar/28.1.27

Kanehisa M. Toward understanding the origin and evolution of cellular organisms. Protein Sci. 2019;28(11):1947-1951. doi:10.1002/pro.3715

Kang R, Zhu S, Zeh HJ, Klionsky DJ, Tang D. BECN1 is a new driver of ferroptosis. Autophagy. 2018;14(12):2173-2175. doi:10.1080/15548627.2018.1513758

Klasson TD, LaGory EL, Zhao H, et al. ACSL3 regulates lipid droplet biogenesis and ferroptosis sensitivity in clear cell renal cell carcinoma. Cancer Metab. 2022;10(1):14. Published 2022 Oct 3. doi:10.1186/s40170-022-00290-z

Kotschi S, Jung A, Willemsen N, et al. NFE2L1-mediated proteasome function protects from ferroptosis. Mol Metab. 2022;57:101436. doi:10.1016/j.molmet.2022.101436

Kwon MY, Park E, Lee SJ, Chung SW. Heme oxygenase-1 accelerates erastin-induced ferroptotic cell death. Oncotarget. 2015;6(27):24393-24403. doi:10.18632/oncotarget.5162

Lee H, Zandkarimi F, Zhang Y, et al. Energy-stress-mediated AMPK activation inhibits ferroptosis. Nat Cell Biol. 2020;22(2):225-234. doi:10.1038/s41556-020-0461-8

Lee JY, Kim WK, Bae KH, Lee SC, Lee EW. Lipid Metabolism and Ferroptosis. Biology (Basel). 2021;10(3):184. Published 2021 Mar 2. doi:10.3390/biology10030184

Lee S, Hwang N, Seok BG, Lee S, Lee SJ, Chung SW. Autophagy mediates an amplification loop during ferroptosis. Cell Death Dis. 2023;14(7):464. Published 2023 Jul 25. doi:10.1038/s41419-023-05978-8

Li D, Li Y. The interaction between ferroptosis and lipid metabolism in cancer. Signal Transduct Target Ther. 2020;5(1):108. Published 2020 Jun 30. doi:10.1038/s41392-020-00216-5

Li J, Cao F, Yin HL, et al. Ferroptosis: past, present and future. Cell Death Dis. 2020;11(2):88. Published 2020 Feb 3. doi:10.1038/s41419-020-2298-2

Li JY, Ren C, Wang LX, et al. Sestrin2 protects dendrite cells against ferroptosis induced by sepsis. Cell Death Dis. 2021;12(9):834. Published 2021 Sep 4. doi:10.1038/s41419-021-04122-8

Li Y, Xu B, Ren X, et al. Inhibition of CISD2 promotes ferroptosis through ferritinophagy-mediated ferritin turnover and regulation of p62-Keap1-NRF2 pathway [published correction appears in Cell Mol Biol Lett. 2023 Aug 24;28(1):69. doi: 10.1186/s11658-023-00478-1]. Cell Mol Biol Lett. 2022;27(1):81. Published 2022 Sep 30. doi:10.1186/s11658-022-00383-z

Li Y, Zhou Y, Liu D, et al. Glutathione Peroxidase 3 induced mitochondria-mediated apoptosis via AMPK /ERK1/2 pathway and resisted autophagy-related ferroptosis via AMPK/mTOR pathway in hyperplastic prostate. J Transl Med. 2023;21(1):575. Published 2023 Aug 26. doi:10.1186/s12967-023-04432-9

Liang J, Liao Y, Wang P, et al. Ferroptosis landscape in prostate cancer from molecular and metabolic perspective. Cell Death Discov. 2023;9(1):128. Published 2023 Apr 15. doi:10.1038/s41420-023-01430-0

Liang P, Xie W, Wang X, et al. Ischemia-inhibited ferric chelate reductase 1 improves ferroptosis-mediated intestinal ischemia injury via Hippo signaling. Int Immunopharmacol. 2024;132:111900. doi:10.1016/j.intimp.2024.111900

Lin C, He J, Tong X, Song L. Copper homeostasis-associated gene PRNP regulates ferroptosis and immune infiltration in breast cancer. PLoS One. 2023;18(8):e0288091. Published 2023 Aug 3. doi:10.1371/journal.pone.0288091

Lin J, Lai Y, Lu F, Wang W. Targeting ACSLs to modulate ferroptosis and cancer immunity. Trends Endocrinol Metab. Published online October 17, 2024. doi:10.1016/j.tem.2024.09.003

Liu J, Kuang F, Kroemer G, Klionsky DJ, Kang R, Tang D. Autophagy-Dependent Ferroptosis: Machinery and Regulation. Cell Chem Biol. 2020;27(4):420-435. doi:10.1016/j.chembiol.2020.02.005

Liu Y, Lu S, Wu LL, Yang L, Yang L, Wang J. The diversified role of mitochondria in ferroptosis in cancer. Cell Death Dis. 2023;14(8):519. Published 2023 Aug 14. doi:10.1038/s41419-023-06045-y

Liuzzi JP, Aydemir F, Nam H, Knutson MD, Cousins RJ. Zip14 (Slc39a14) mediates non-transferrin-bound iron uptake into cells. Proc Natl Acad Sci U S A. 2006;103(37):13612-13617. doi:10.1073/pnas.0606424103

Luo L, Zhang Z, Weng Y, Zeng J. Ferroptosis-Related Gene GCLC Is a Novel Prognostic Molecular and Correlates with Immune Infiltrates in Lung Adenocarcinoma. Cells. 2022;11(21):3371. Published 2022 Oct 25. doi:10.3390/cells11213371

Miess H, Dankworth B, Gouw AM, et al. The glutathione redox system is essential to prevent ferroptosis caused by impaired lipid metabolism in clear cell renal cell carcinoma. Oncogene. 2018;37(40):5435-5450. doi:10.1038/s41388-018-0315-z

Mishima E. The E2F1-IREB2 axis regulates neuronal ferroptosis in cerebral ischemia. Hypertens Res. 2022;45(6):1085-1086. doi:10.1038/s41440-021-00837-5

Oh M, Jang SY, Lee JY, et al. The lipoprotein-associated phospholipase A2 inhibitor Darapladib sensitises cancer cells to ferroptosis by remodelling lipid metabolism. Nat Commun. 2023;14(1):5728. Published 2023 Sep 15. doi:10.1038/s41467-023-41462-9

Ou Y, Wang SJ, Li D, Chu B, Gu W. Activation of SAT1 engages polyamine metabolism with p53-mediated ferroptotic responses. Proc Natl Acad Sci U S A. 2016;113(44):E6806-E6812. doi:10.1073/pnas.1607152113

Park SJ, Cho SS, Kim KM, et al. Protective effect of sestrin2 against iron overload and ferroptosis-induced liver injury. Toxicol Appl Pharmacol. 2019;379:114665. doi:10.1016/j.taap.2019.114665

Pearson SA, Cowan JA. Evolution of the human mitochondrial ABCB7 [2Fe-2S](GS)4 cluster exporter and the molecular mechanism of an E433K disease-causing mutation. Arch Biochem Biophys. 2021;697:108661. doi:10.1016/j.abb.2020.108661

Peng B, Peng J, Kang F, Zhang W, Peng E, He Q. Ferroptosis-Related Gene MT1G as a Novel Biomarker Correlated With Prognosis and Immune Infiltration in Colorectal Cancer. Front Cell Dev Biol. 2022;10:881447. Published 2022 Apr 20. doi:10.3389/fcell.2022.881447

Qi L, Sun B, Yang B, Lu S. PGM5P3-AS1 regulates MAP1LC3C to promote cell ferroptosis and thus inhibiting the malignant progression of triple-negative breast cancer. Breast Cancer Res Treat. 2022;193(2):305-318. doi:10.1007/s10549-021-06501-3

Ru Q, Li Y, Chen L, Wu Y, Min J, Wang F. Iron homeostasis and ferroptosis in human diseases: mechanisms and therapeutic prospects. Signal Transduct Target Ther. 2024;9(1):271. Published 2024 Oct 14. doi:10.1038/s41392-024-01969-z

Ryan SK, Zelic M, Han Y, et al. Microglia ferroptosis is regulated by SEC24B and contributes to neurodegeneration. Nat Neurosci. 2023;26(1):12-26. doi:10.1038/s41593-022-01221-3

Santana-Codina N, Gikandi A, Mancias JD. The Role of NCOA4-Mediated Ferritinophagy in Ferroptosis. Adv Exp Med Biol. 2021;1301:41-57. doi:10.1007/978-3-030-62026-4_4

Schneider C, Hilbert J, Genevaux F, et al. A Novel AMPK Inhibitor Sensitizes Pancreatic Cancer Cells to Ferroptosis Induction. Adv Sci (Weinh). 2024;11(31):e2307695. doi:10.1002/advs.202307695

Shin D, Lee J, You JH, Kim D, Roh JL. Dihydrolipoamide dehydrogenase regulates cystine deprivation-induced ferroptosis in head and neck cancer. Redox Biol. 2020;30:101418. doi:10.1016/j.redox.2019.101418

Sorets AG, Rosch JC, Duvall CL, Lippmann ES. Caveolae-Mediated Transport at the Injured Blood-Brain Barrier as an Underexplored Pathway for Central Nervous System Drug Delivery. Curr Opin Chem Eng. Dec 2020;30:86-95

Sousa JA, Callejas BE, Wang A, et al. GPx1 deficiency confers increased susceptibility to ferroptosis in macrophages from individuals with active Crohn's disease. Cell Death Dis. 2024;15(12):903. Published 2024 Dec 18. doi:10.1038/s41419-024-07289-y

Sun J, Zhou C, Zhao Y, et al. Quiescin sulfhydryl oxidase 1 promotes sorafenib-induced ferroptosis in hepatocellular carcinoma by driving EGFR endosomal trafficking and

inhibiting NRF2 activation. Redox Biol. 2021;41:101942. doi:10.1016/j.redox.2021.101942

Sun K, Zhi Y, Ren W, et al. The mitochondrial regulation in ferroptosis signaling pathway and its potential strategies for cancer. Biomed Pharmacother. 2023;169:115892. doi:10.1016/j.biopha.2023.115892

Sun X, Ou Z, Chen R, et al. Activation of the p62-Keap1-NRF2 pathway protects against ferroptosis in hepatocellular carcinoma cells. Hepatology. 2016;63(1):173-184. doi:10.1002/hep.28251

Sun X, Ou Z, Xie M, et al. HSPB1 as a novel regulator of ferroptotic cancer cell death. Oncogene. 2015;34(45):5617-5625. doi:10.1038/onc.2015.32

Tang D, Chen X, Kang R, Kroemer G. Ferroptosis: molecular mechanisms and health implications. Cell Res. 2021;31(2):107-125. doi:10.1038/s41422-020-00441-1

Tang W, Xu F, Zhao M, Zhang S. Ferroptosis regulators, especially SQLE, play an important role in prognosis, progression and immune environment of breast cancer. BMC Cancer. 2021;21(1):1160. Published 2021 Oct 29. doi:10.1186/s12885-021-08892-4

Thorwald MA, Godoy-Lugo JA, Garcia G, et al. Iron-associated lipid peroxidation in Alzheimer's disease is increased in lipid rafts with decreased ferroptosis suppressors, tested by chelation in mice. Alzheimers Dement. 2025;21(1):e14541. doi:10.1002/alz.14541

Torres-Velarde JM, Allen KN, Salvador-Pascual A, et al. Peroxiredoxin 6 suppresses ferroptosis in lung endothelial cells. Free Radic Biol Med. 2024;218:82-93. doi:10.1016/j.freeradbiomed.2024.04.208

Wang H, Li Y, Liu X, Wu Y. Identification and validation of ferroptosis-related gene SLC2A1 as a novel prognostic biomarker in AKI. Aging (Albany NY). 2024;16(6):5634-5650. doi:10.18632/aging.205669

Wang S, Wu C, Ma D, Hu Q. Identification of a ferroptosis-related gene signature (FRGS) for predicting clinical outcome in lung adenocarcinoma. PeerJ. 2021;9:e11233. Published 2021 Apr 13. doi:10.7717/peerj.11233

Xia Y, Liu S, Li C, et al. Discovery of a novel ferroptosis inducer-talaroconvolutin A-killing colorectal cancer cells in vitro and in vivo. Cell Death Dis. 2020;11(11):988. Published 2020 Nov 17. doi:10.1038/s41419-020-03194-2

Yan R, Lin B, Jin W, Tang L, Hu S, Cai R. NRF2, a Superstar of Ferroptosis. Antioxidants (Basel). 2023;12(9):1739. Published 2023 Sep 8. doi:10.3390/antiox12091739

Yang WH, Lin CC, Wu J, et al. The Hippo Pathway Effector YAP Promotes Ferroptosis via the E3 Ligase SKP2. Mol Cancer Res. 2021;19(6):1005-1014. doi:10.1158/1541-7786.MCR-20-0534

Yang Y, Wang Y, Guo L, Gao W, Tang TL, Yan M. Interaction between macrophages and ferroptosis. Cell Death Dis. 2022;13(4):355. Published 2022 Apr 16. doi:10.1038/s41419-022-04775-z

Yang Z, Su W, Wei X, et al. HIF-1α drives resistance to ferroptosis in solid tumors by promoting lactate production and activating SLC1A1. Cell Rep. 2023;42(8):112945. doi:10.1016/j.celrep.2023.112945

Yao F, Cui X, Zhang Y, et al. Iron regulatory protein 1 promotes ferroptosis by sustaining cellular iron homeostasis in melanoma. Oncol Lett. 2021;22(3):657. doi:10.3892/ol.2021.12918

Ye L, Wen X, Qin J, et al. Metabolism-regulated ferroptosis in cancer progression and therapy. Cell Death Dis. 2024;15(3):196. Published 2024 Mar 8. doi:10.1038/s41419-024-06584-y

You L, Yu PP, Dong T, et al. Astrocyte-derived hepcidin controls iron traffic at the blood-brain-barrier via regulating ferroportin 1 of microvascular endothelial cells. Cell Death Dis. Aug 1 2022;13(8):667. doi:10.1038/s41419-022-05043-w

Yuan H, Li X, Zhang X, Kang R, Tang D. CISD1 inhibits ferroptosis by protection against mitochondrial lipid peroxidation. Biochem Biophys Res Commun. 2016;478(2):838-844. doi:10.1016/j.bbrc.2016.08.034

Zhan M, Ding Y, Huang S, et al. Lysyl oxidase-like 3 restrains mitochondrial ferroptosis to promote liver cancer chemoresistance by stabilizing dihydroorotate dehydrogenase. Nat Commun. 2023;14(1):3123. Published 2023 May 30. doi:10.1038/s41467-023-38753-6

Zhang T, Wang S, Hua D, et al. Identification of ZIP8-induced ferroptosis as a major type of cell death in monocytes under sepsis conditions. Redox Biol. 2024;69:102985. doi:10.1016/j.redox.2023.102985

Zhang W, Huang F, Ding X, Qin J, Wang W, Luo L. Identifying ALOX15-initiated lipid peroxidation increases susceptibility to ferroptosis in asthma epithelial cells. Biochim Biophys Acta Mol Basis Dis. 2024;1870(5):167176. doi:10.1016/j.bbadis.2024.167176

Zhang Y, Koppula P, Gan B. Regulation of H2A ubiquitination and SLC7A11 expression by BAP1 and PRC1. Cell Cycle. 2019;18(8):773-783. doi:10.1080/15384101.2019.1597506

Zheng S, Mo J, Zhang J, Chen Y. HIF-1α inhibits ferroptosis and promotes malignant progression in non-small cell lung cancer by activating the Hippo-YAP signalling pathway. Oncol Lett. 2023;25(3):90. Published 2023 Jan 19. doi:10.3892/ol.2023.13676

Zhou L, Yang C, Zhong W, et al. Chrysin induces autophagy-dependent ferroptosis to increase chemosensitivity to gemcitabine by targeting CBR1 in pancreatic cancer cells. Biochem Pharmacol. 2021;193:114813. doi:10.1016/j.bcp.2021.114813

Zhu S, Zhang Q, Sun X, et al. HSPA5 Regulates Ferroptotic Cell Death in Cancer Cells. Cancer Res. 2017;77(8):2064-2077. doi:10.1158/0008-5472.CAN-16-1979

Zou Y, Palte MJ, Deik AA, et al. A GPX4-dependent cancer cell state underlies the clear-cell morphology and confers sensitivity to ferroptosis. Nat Commun. 2019;10(1):1617. Published 2019 Apr 8. doi:10.1038/s41467-019-09277-9
