# Supplementary material for: Ferroptosis in microglial activation: a systematic review and multidata comparison
Source: Brain Commun. 2026 Mar 30;8(2):fcag109. doi: 10.1093/braincomms/fcag109 (PMC13056721; doi:10.1093/braincomms/fcag109)
Supplement: fcag109_Supplementary_Data [file fcag109_supplementary_data.zip › Supplementary_Table_Headings.pdf]

Supplementary table 1. List of ferroptosis-associated proteins.

Our manually generated list of 120 ferroptosis-relevant proteins, providing 78 unique entries (pink) compared to the commonly used KEGG pathway containing 42 entries (orange) (source: KEGG, https://www.genome.jp/entry/pathway+hsa04216; November 28, 2025). As separate columns contain brief descriptions relevant to ferroptosis, accompanied by a column with the respective reference.

Supplementary table 2. List of abbreviations.
